# Expression and structural analysis of taste receptor genes in Iberian and Duroc pigs

**DOI:** 10.1186/s12711-025-00968-0

**Published:** 2025-05-02

**Authors:** Cristina Óvilo, Rita Benítez, Yolanda Núñez, Ramón Peiró-Pastor, Fabian García, Eduardo De Mercado, Emilio Gómez-Izquierdo, Juan García-Casco, Clemente López-Bote, María Muñoz

**Affiliations:** 1 Departamento de Mejora Genética Animal, INIA-CSIC, Madrid, Spain; 2 Departamento de Producción Animal, Facultad de Veterinaria, Universidad Complutense de Madrid, Madrid, Spain; 3 Centro de Pruebas de Porcino ITACYL, Hontalbilla, Segovia Spain

## Abstract

**Background:**

Taste receptor genes are expressed in sensory cells located in the tongue and influence food preferences, voluntary feed intake, and other relevant traits. Taste perception may differ between livestock breeds that show differences in eating behaviour and between animals that receive different diets or show phenotypic variation in feed intake or related-traits. The objectives of this work were to deepen the understanding of the regulation of the function of taste receptor genes in the circumvallate papillae of obese Iberian pigs in comparison to Duroc pigs, and to characterize their genetic variation and associations with relevant production traits.

**Results:**

We performed a gene expression and structural analysis of ten taste receptor genes in Iberian and Duroc pigs. Gene expression was quantified in the circumvallate papillae of 48 growing Iberian and Duroc pigs maintained under identical management conditions but fed isocaloric diets differing in energy source: either high concentration of fat rich in oleic acid (HO) or carbohydrates (CH); and sacrificed after 47 days of treatment (50.5 kg live weight). Gene expression differed between the two breeds for most of the analyzed genes, with the *TAS1R1, TAS1R2, TAS1R3*, *TAS2R4*, *TAS2R38*, *TAS2R39*, *GPR84,* and *CD36* genes being overexpressed in Duroc pigs. The diet effect was modulated by breed, with *TAS1R1*, *TAS1R3,* and *TAS2R4* genes being overexpressed only in Duroc pigs fed the HO diet. Detection of genetic variants (single nucleotide polymorphisms, SNPs) for this panel of genes was performed on muscle RNA-seq data, and three SNPs in the *TAS1R1*, *TAS1R3,* and *CD36* genes were selected for association studies. All three SNPs were associated with various growth, fattening, tissue fat content, and composition traits. Moreover, the *CD36:c.910G/T* SNP was associated with oral *CD36* gene expression and with differences in the predicted mRNA secondary structure.

**Conclusions:**

Most taste receptor genes are expressed at lower level in circumvallate papillae from Iberian than Duroc pigs. This aligns with lower overall taste sensitivity, higher feed intake, and obese nature of Iberian pigs. Significant association results were observed for SNPs in the *TAS1R1* and *TAS1R3* genes with meat quality traits and liver composition, which showed segregation in world-wide distributed breeds, but particularly for a potential causal SNP in the *CD36* gene, associated with growth and tissue composition, which segregates in Iberian populations.

**Supplementary Information:**

The online version contains supplementary material available at 10.1186/s12711-025-00968-0.

## Background

The Iberian pig is a traditional breed raised in the southwestern Iberian Peninsula and is characterized by slow growth and high intramuscular fat, which contributes to its uniquely desired flavor [1]. This breed differs from world-wide distributed pig breeds in terms of metabolism and many relevant phenotypic traits [2]. In Iberian pig production, crossbreeding with Duroc sires is a common practice to enhance growth efficiency. Large differences in composition and meat quality traits have been reported between Iberian pork and pork from common lean industrial crosses [3–5], and some differences have also been found between Iberian and Iberian × Duroc dry-cured hams [6]. The main phenotypic and metabolic differences between Iberian and other pig breeds are attributed to the characteristic thrifty metabolism of Iberian pigs [7]. These features result from their long adaptation to the challenging environmental conditions of their traditional outdoor production system [8]. Iberian pigs accumulate energy reserves in periods of abundant food to prepare for times of scarcity, which has been associated with a leptin-resistant state. Thus, Iberian pigs show deregulation of the hypothalamic circuitry that controls voluntary feed intake and energy balance, resulting in an elevated appetite. This leptin resistance is in part explained by a mutation in the leptin receptor gene, which is fixed in the Iberian pig, but segregates in other breeds [9]. Nevertheless, other pathways involved in eating behavior have yet to be explored in this breed.

The gustatory system in mammals comprises taste receptor cells (TRCs) that are organized in taste buds within gustatory papillae, mostly located in the tongue [10, 11]. These TRCs express taste receptor genes, which encode chemoreceptors that detect dietary compounds in the oral cavity, generating signals that are transmitted to the brain, thus leading to behavioral responses to taste stimuli [12]. The main receptors related to nutrient sensing belong to the family of G-protein coupled receptors (GPCRs), which are encoded by several genes that are mainly grouped into two families. TAS1R genes are related to the perception of simple sugars and some L-amino acids present in the diet (sweet and umami taste perception), with *TAS1R1* and *TAS1R3* genes sensing amino acids and *TAS1R2* and *TAS1R3* sensing sugars. TAS2R genes are involved in the sensory mechanism that identifies potentially toxic compounds (bitter, acrid, or astringent taste perception). Other GPCRs related to nutrient sensing in the oral cavity include those involved in the sensing of amino acids and peptones, as well as medium- and long-chain saturated and unsaturated fatty acids, such as CD36, GPR40, GPR41, GPR43, GPR84, and GPR120 [13]. In addition to the tongue and oral cavity, taste receptors are also known to play relevant roles in other organs, especially in the gastrointestinal tract, through endocrine mechanisms [14, 15].

Taste perception is associated with feeding behavior that may affect voluntary feed intake and potentially influences relevant traits related to productive efficiency, energy balance, and meat quality. However, there are limited studies on the expression, diet regulation, structural variation, and associations of taste receptor genes with production traits in livestock animals. Clop et al*.* [16] reported a potential relationship of some of taste receptor genes with growth and fat deposition, which were attributed to changes in taste preferences, appetite, or reward behaviour. Further validation studies from Cirera et al. [17] confirmed some of these results and showed an enrichment in genetic associations with obesity-related phenotypes for genetic markers that were selected based on previous information based on their predicted impact on protein function, compared to the use of a standard set of markers included in a commercial genotyping array. Fontanesi et al*.* [18] reported an association between variation in the *GPR120* gene and growth rate, while Ribani et al*.* [19] found that variants in the *TAS2R38* and *TAS2R39* genes were associated with backfat thickness in Italian Large White pigs. Moreover, *TAS2R38* was also proposed as a positional candidate gene for body weight and fatness in a genome-wide association study [20]. In humans, variants in many different taste receptor genes have been related to eating behavior, body weight at different developmental stages, and a number of diet-related health problems such as obesity, diabetes, and cancer [21–23].

This study hypothesizes that gene expression of taste receptor genes may differ between breeds that have distinguishable eating behaviors, as well as between animals that receive different diets. Potential differences in gene expression between breeds may be connected to *cis* structural variation. Therefore, the study of the structure, function, and regulation of taste receptor genes, including their expression and genetic variation, could contribute to improving our understanding of the genetic basis of relevant productive traits related to voluntary food intake and energy balance. Thus, the objective of this study was to explore the level of expression of a panel of taste receptor genes in the taste buds of Iberian and Duroc purebred pigs fed diets with different energy sources. We also aimed to investigate genetic variants in these genes and their associations with productive traits in a commercial crossbred Duroc x Iberian pig population.

## Methods

### Animals and sampling

The animals used for the gene expression study were obtained from an experiment performed at the Pig Test Center ITACYL (Instituto Tecnológico Agrario de Castilla y León, Hontalbilla, Segovia, Spain), as previously described [24, 25]. Briefly, the experiment included a total of 48 animals, 29 Iberian (Torbiscal strain) and 19 Duroc males, born in 32 contemporary litters at the same farm. At 19.9 ± 3.8 kg of live weight (LW) and 10 (± 1.6 days) weeks of age, the animals were allocated to two experimental groups that were fed different isocaloric and isoproteic diets (13.9 MJ digestible energy/kg and 156 g crude protein/kg) in a factorial (2 breed × 2 diets) experiment design. The two diets contained either a high concentration of fat rich in oleic acid (HO) or high concentrations of carbohydrates (CH). Details on experimental diets can be found in Additional file 1 Table S1. Diets were analysed for dry matter (method 930.15) and crude fat (method 920.39), as proposed by AOAC International [26], and fatty acids concentrations were quantified following the procedure described by Sukhija and Palmquist [27]. Feed and fresh water were provided for ad libitum consumption. Pigs were kept under identical management conditions and were housed in 20 boxes (3–5 pigs/pen, avoiding the inclusion of littermates in the same pen), until slaughter after forty-seven days on the treatment diets, at LWs of 50.2 ± 7.5 and 51.2 ± 5.09 kg for the Iberian and Duroc pigs, respectively. The animals (29 Iberian pigs: HO diet n = 16, CH diet n = 13; 19 Duroc pigs: HO diet n = 10, CH diet n = 9) were sampled immediately after euthanasia by stunning and exsanguination, in compliance with RD 53/2013 standard procedures. Circumvallate papillae were sampled from all animals for gene expression studies by using a scalpel to gently remove the two circumvallate papillae of each animal, located in the caudal region of the dorsal surface of the tongue and surrounded by a deep circular groove [11]. Biceps femoris muscle was also sampled from each animal for muscle tissue composition and transcriptome analyses (methodological details and results reported in [24]). All samples were kept in liquid N_2_ and stored at − 80 °C until use.

For the genetic association studies, DNA samples and phenotypic data from a commercial Iberian x Duroc crossbred population were used (n = 384). Details on breeding, management and phenotype measurements are described in a previous work [28], and a list of the recorded traits can be found in Additional file 2 Table S2.

### Quantification of gene expression

A total of 10 taste receptor genes (*TAS1R1, TAS1R2, TAS1R3, TAS2R4, TAS2R38, TAS2R39, GPR40, GPR84, GPR120,* and *CD36*) were selected for the gene expression study, as they have been reported to be key components in the sensing of sugars, aminoacids, bitterness, umami, or fatty acids, and with previous works reporting structural variation and genetic association with relevant traits [19, 29, 30]. Total RNA was isolated from the available papillae samples (50–100 mg) from each animal using the RiboPureTM RNA isolation kit (Ambion, Austin, TX, USA), following the manufacturer’s recommendations. The obtained RNA was quantified using NanoDrop equipment (NanoDrop Technologies, Wilmington, DE, USA) and the RNA quality was assessed with an Agilent 2100 bioanalyzer device (Agilent Technologies, Palo Alto, CA, USA).

The expression of the 10 candidate taste receptor genes was quantified by RT‒qPCR. First-strand cDNA synthesis was carried out with SuperScript III (Invitrogen, Life Technologies, Paisley, UK) and random hexamers in a total volume of 20 μL containing 1 μg of total RNA, following the supplier’s instructions. Primers were designed for each gene, covering different exons to ensure amplification of the cDNA, using Primer Select Software (DNASTAR, Madison, WI, USA) from the available ENSEMBL sequences. The specific primer sequences used are shown in Additional file 3 Table S3. Gene expression was quantified using *GAPDH* and *ACTB* as housekeeping genes, which were determined to be the two most stable endogenous genes for data normalization from among *GAPDH, ACTB, TBP, 18S, PPIA,* and *B2M*, using the Genorm software [31]. Transcript quantification was performed using SYBR Green mix (Roche, Rotkreuz, Switzerland) in a LightCycler 480 II (Roche, Rotkreuz, Switzerland).

### Identification of SNPs

Muscle transcriptome data previously obtained from the same experimental animals [24] were employed in the search for polymorphisms within the 10 candidate taste receptor genes. For preprocessing of alignment files, a sequence dictionary was created, duplicated reads were marked, read groups were added, and bam files were sorted and indexed with Picard Tools 8 (http://broadinstitute.github.io/picard/). Variant calling analysis of preprocessed alignment files was performed using the Genome Analysis Toolkit (GATK, version 4.2.6.1) [32], with identification of potential variants in each sample using the HaplotypeCaller tool. A filter was applied to remove sites with a higher probability of being artifacts with the VariantFiltration tool. VCF files corresponding to the same experimental group were combined into a single file with the CombineGVCFs tool. The GenotypeGVCF tool was used to convert gvcf to vcf. Variant calling was then completed separately for each sample using HaplotypeCaller in -ERC GVCF mode, leveraging the previously introduced reference model [32] to produce a comprehensive record of genotype likelihoods and annotations for each site in the genome in the form of a gVCF file (genomic VCF). In a second step, a joint genotyping analysis of the gVCF files produced for all samples in the cohort was done. The numbers of variants by type and by experimental group were obtained using vcfstats.

### Genotyping

Six SNPs located in the *TAS1R1*, *TAS1R3,* and *CD36* genes (two SNPs in each gene that showed different segregation patterns and that were adequate for KASP genotyping) were selected for genotyping in several Iberian and Duroc pig populations. Genotyping of the six SNPs was performed by KASP, a Kompetitive Allele Specific PCR genotyping system (LGC Biosearch Technologies, Hoddeston, Herts, United Kingdom), on the 29 Iberian and 19 Duroc pigs that were employed for the gene expression study and on 52 Iberian x Duroc crossbred animals from a commercial population with relevant phenotypic data available. The specific oligos for the alleles of each SNP under study were designed with Kraken software (LGC Biosearch Technologies, Hoddeston, Herts, United Kingdom). Each KASP reaction was set up in a final volume of 5 µl, including 5–50 ng cDNA from papillae tissue (for the 48 purebred animals) or DNA (for the 52 Duroc x Iberian crossbreds), KASP assay mix (containing two competitive allele-specific primers, one for each SNP allele, and a common reverse primer), and KASP master mix (FRET cassette plus Taq polymerase in an optimized buffer solution). Genotyping was performed in a LightCycler 480II (Roche, Rotkreuz, Switzerland).

For genetic association studies, three of the six SNPs were selected for genotyping in a broader commercial population of Duroc x Iberian crossbred pigs with relevant phenotypic data available (n = 384, including the previous 52 crossbreds) [28]. For allele frequency estimation, these three SNPs were also genotyped in a panel of pig samples (n = 97) from different world-wide distributed pure breeds (Duroc, Pietrain, Landrace, Large White) and European and Asiatic wild boars. DNA extraction was performed from blood samples using the NucleoSpin® Blood Kit (Macherey–Nagel, Düren, Germany).

### Statistical analyses

#### Influence of breed and diet on gene expression

The influence of diet and breed on the expression of candidate genes was analyzed with a linear model fitting breed, diet, qPCR plate, and breed × diet as fixed effects and litter and box as random effects, following the method proposed by Steibel et al. [33]. Analyses were performed with the MIXED procedure of SAS 9.4 (SAS Inst. Inc., Cary, NC, EE.UU). The raw correlations of the normalized gene expression data among the different tested genes and with the phenotypic data was studied within each breed in the R environment [34]. The following phenotypes were analyzed: muscle fatty acid composition, feed intake, backfat thickness at the loin and ham levels, intramuscular fat percentage of the l. dorsi and b. femoris muscles, adipocyte size, and body weight at slaughter [24]. Multiple test corrections using Benjamin and Hochberg false discovery rate (FDR) were carried out [35].

#### Association of SNPs within candidate genes with productive traits and gene expression

Association analyses with phenotype and gene expression data were performed for three SNPs, one selected for each candidate gene showing polymorphism. The *TAS1R1* gene c.405G/C was selected because it was the only marker showing segregation in the crossbred population based on the genotypes of the initial 52 animals. For the *TAS1R3* and *CD36* genes, the two SNPs analyzed in each gene were cosegregating, and the c.-2126A/G and c.910G/T SNPs were selected for the respective genes.

For analysis of associations of SNP genotypes with phenotypes, the statistical model shown in Eq. (1) was used:

1 $$\mathbf{y}=\text{ a}+\mathbf{b}\mathbf{X}+\mathbf{f}\mathbf{F}+\mathbf{g}+\text{e},$$

where **y** represents the vector of phenotypic values of a given trait, **a** denotes the mean term, **b** encompasses the additive effect (fixed term) of the analyzed SNP (*TAS1R1*:c.405G/C, *TAS1R3*: c.-2126A/G and *CD36:*c.910G/T), **X** is the vector of the genotype covariate for one of the SNPs (fitted one at a time), coded as 0, 1, or 2, **f** includes the remaining fixed effects and covariates, which differed depending on the trait analyzed and are specified in Additional file 4 Table S4 [28], and **F** stands for the corresponding incidence matrix. In addition, **g** is the accumulated effect of all other SNPs captured by the genomic relationship matrix (GRM), which was built using the available genotyping data for 1005 SNPs obtained in our previous study for the same animal population [28], along with the three SNPs (*TAS1R1*:c.405G/C, *TAS1R3*: c.-2126A/G and *CD36:*c.910G/T) studied in the present work. The method MLMA-LOCO was used, in which the GRM is constructed separately for each chromosome by excluding the SNPs located on the chromosome currently being tested for association. These analyses were carried out with the GCTA software [36].

The effect of each of the three selected SNPs on the expression of the corresponding candidate genes was also evaluated using the normalized gene expression data from the circumvallate papillae of the pure Iberian and Duroc populations. Analyses were performed within breed, studying for each gene the breed in which segregation of the SNP was observed (*TAS1R1* and *TAS1R3* in Duroc and *CD36* in Iberian), using the linear model included in Eq. (2). Box and litter effects were excluded after preliminary analyses in which no significant effects were found for these factors.

2 $$\mathbf{y} =\text{ Mean }+ \mathbf{S}\mathbf{N}\mathbf{P}\, \mathbf{G}\mathbf{e}\mathbf{n}\mathbf{o}\mathbf{t}\mathbf{y}\mathbf{p}\mathbf{e} + \mathbf{D}\mathbf{i}\mathbf{e}\mathbf{t} +\text{ e},$$

The effects of genotype and diet on gene expression were analyzed as fixed effects. These analyses were conducted using the *lm* and *emmeans* functions in R.

#### In silico prediction of the mRNA secondary structure, protein secondary structure, and microRNA binding sites for the CD36 gene

The potential effect of CD36c.910G/T on mRNA folding was predicted using the RNAfold tool, which is available at the Vienna RNA website [37]. Both alleles were analyzed for potential folding and changes in minimum free energy secondary structures were evaluated. PSI-blast based secondary structure PREDiction (PSIPRED) [38] was used to evaluate if the aminoacidic change could cause a change in the conformation of the secondary structure of the protein, thus altering its functionality. In addition, the change in a microRNA binding site in the 3’UTR of *CD36* due to the presence of the CD36:c.1255T/C SNP (which cosegregates with CD36:c.910G/T) was assessed using MicroRNA Target Prediction Database (mirDB) with the *Custom Prediction* tool [39], which searches for miRNA seed binding sites and generates targeting features for MirTarget prediction. This search was conducted using information of human miRNA, as pig miRNAs were not available in this database.

## Results

### Candidate gene expression: effects of breed and diet

**Fig. 1:**
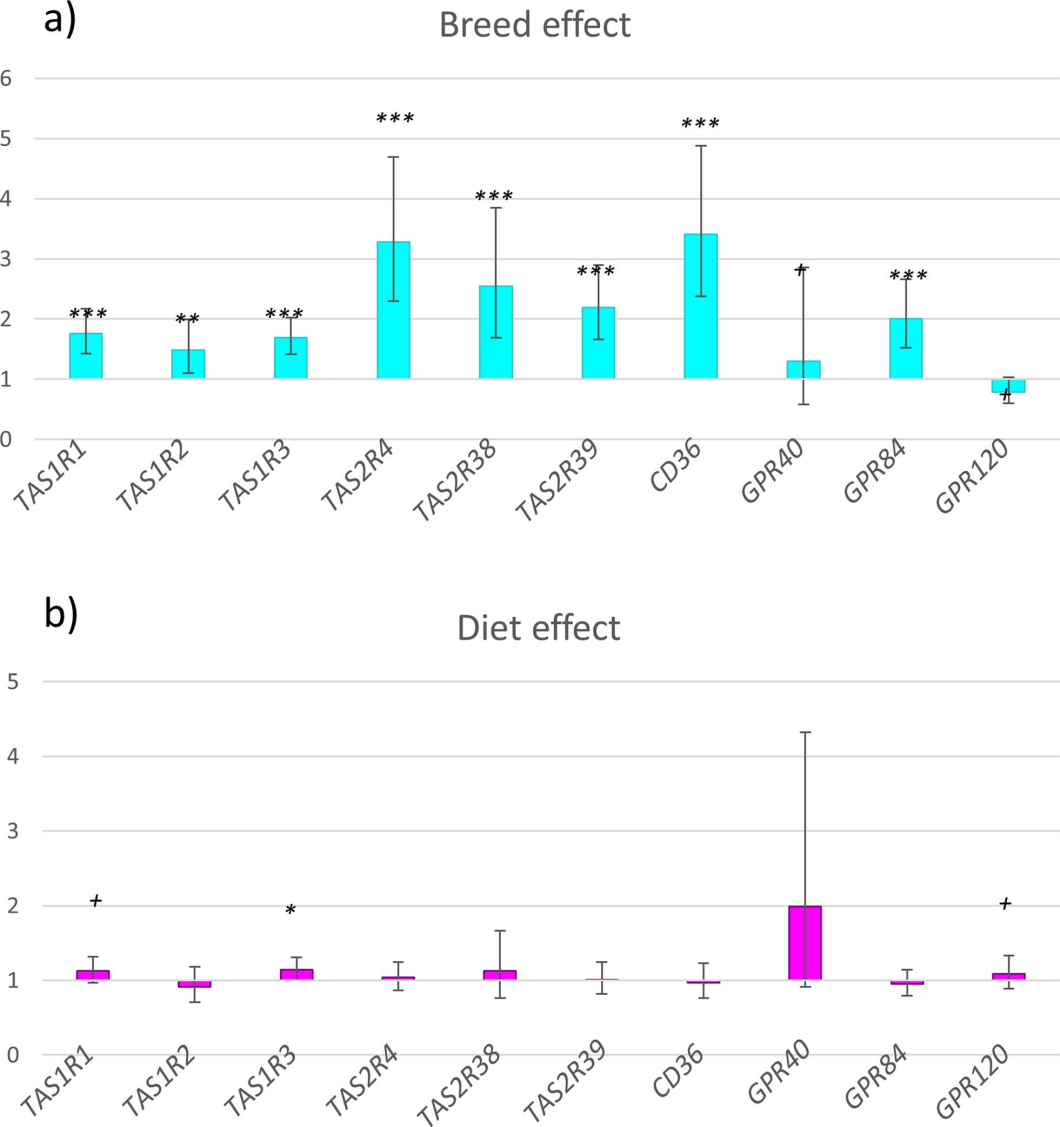
Effects of diet and breed on the expression of taste receptor genes. Fold change in the relative expression of candidate genes according to the two tested factors. **a** Breed effect; values higher than 1 indicate higher expression in Duroc pigs. **b** Diet effect; values higher than 1 indicate greater expression in HO diet group. P value: +  < 0.10; * < 0.05; ** < 0.01; *** < 0.001

Results of the comparison of the expression of candidate genes between breeds and diets are shown in Fig. 1. All six analyzed taste receptor genes from the TAS1R and TAS2R gene families (*TAS1R1, TAS1R2, TAS1R3, TAS2R4, TAS2R38* and *TAS2R39*) were overexpressed in the Duroc breed. Two genes that encode fatty acid receptors (*GPR84* and *CD36*) were also upregulated in Duroc compared with Iberian samples and a third gene showed a trend in the same direction (*GPR40*, p value = 0.07). Only *GPR120* was suggestively overexpressed in Iberian papillae (p value = 0.06).

The effect of diet on gene expression was small, affecting only *TAS1R3*, which was overexpressed in HO (p value = 0.05).

**Fig. 2:**
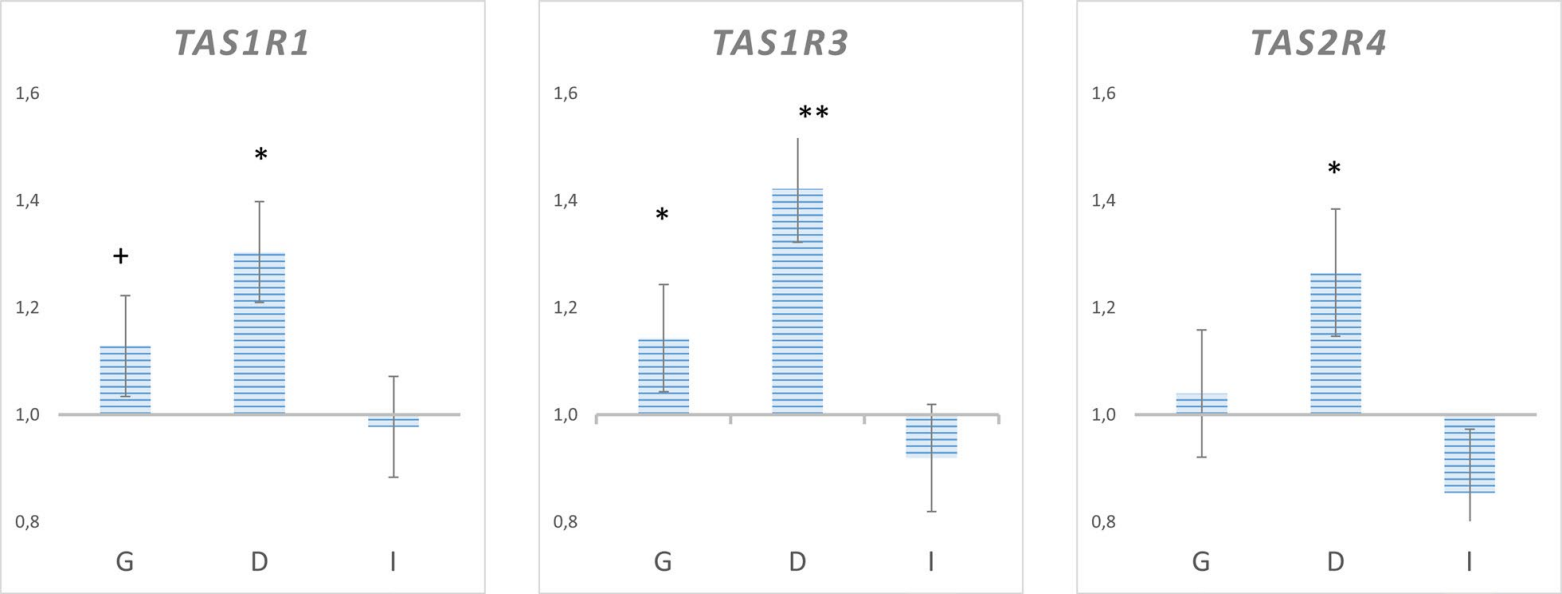
Breed x diet interaction effects on relative gene expression. Figures show the fold change in gene expression according to diet in different breed groups (values higher than 1 indicate higher expression in HO): G (global population: Duroc + Iberians), D (Duroc), I (Iberian), P value: +  < 0.10; * < 0.05; ** < 0.01

Significant breed x diet interactions were detected for the *TAS1R3* and *TAS2R4* genes (p values = 0.002 and 0.03, respectively). A similar pattern was observed for *TAS1R1,* but without statistical significance (p value = 0.06). In all cases, the interactions indicated that the upregulation of these three genes in the HO diet was only significant in the Duroc breed (Fig. 2).

### Correlation of gene expression and phenotypic data

**Fig. 3:**
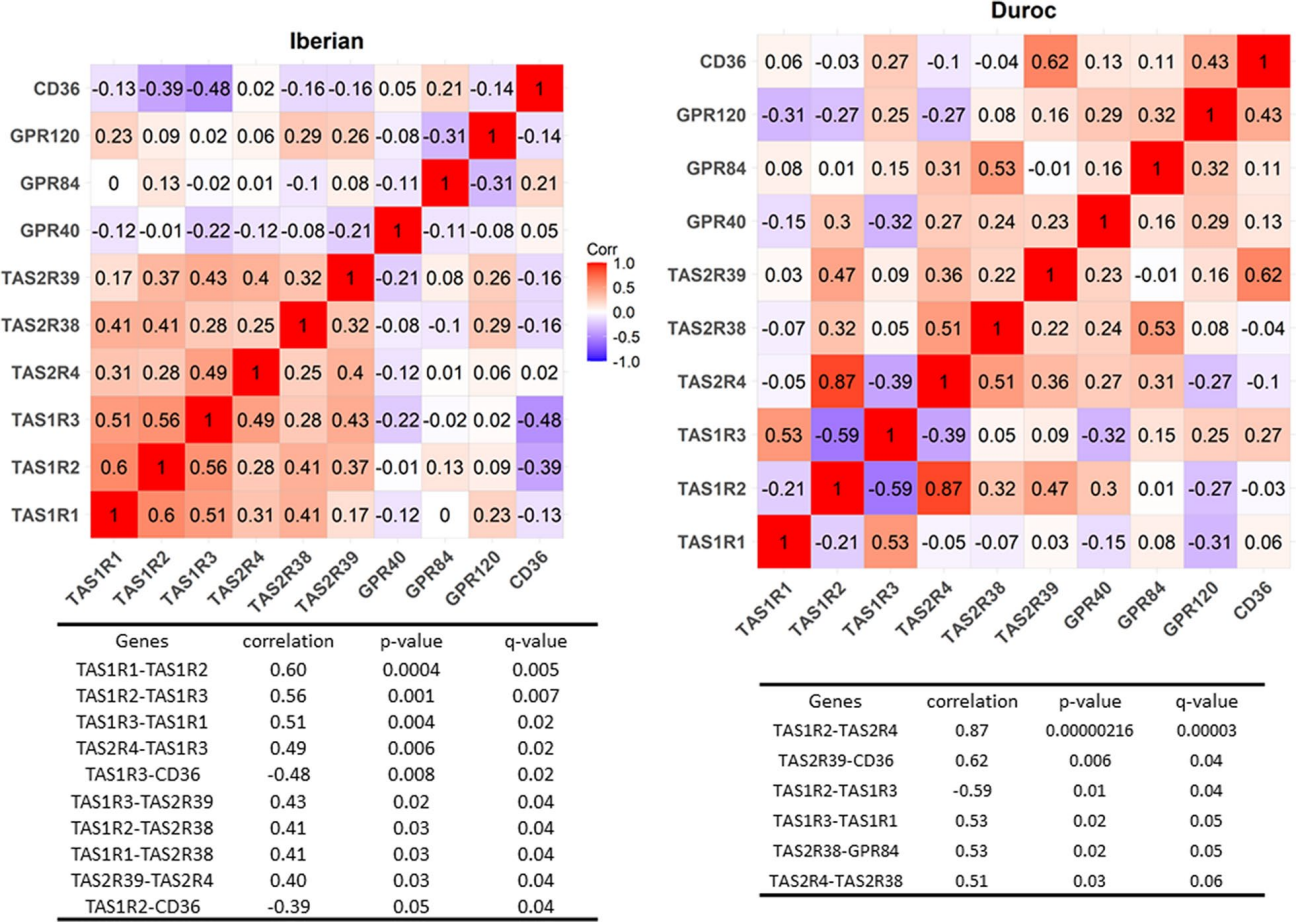
Correlation of the levels of expression of taste receptor genes. Correlations of normalized gene expression data between all pairs of analyzed genes in the Iberian and Duroc breeds. The tables below the figures include correlations significantly different from zero after multiple test correction (q < 0.05)

**Fig. 4:**
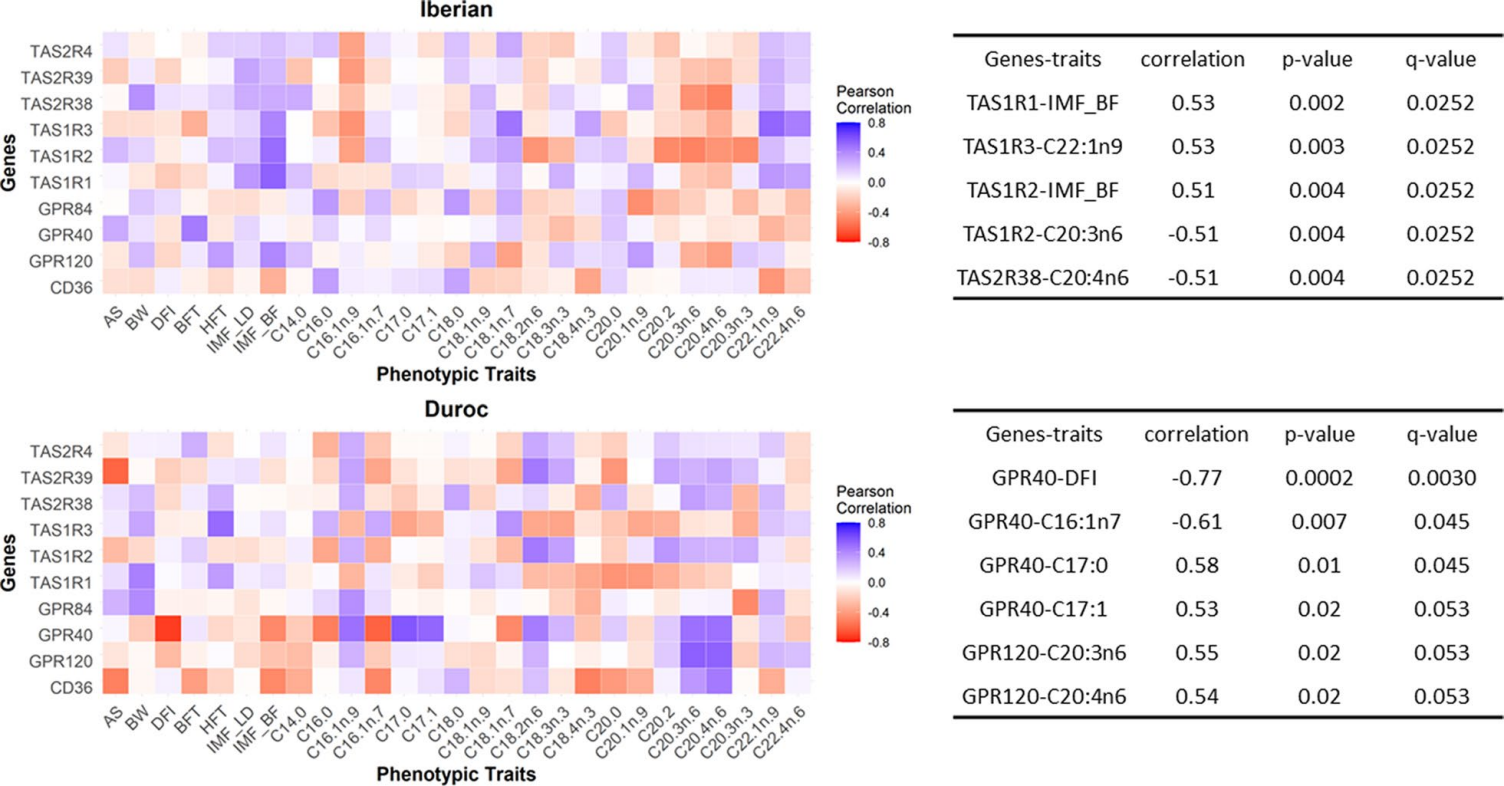
Correlations of levels of expression of taste receptor genes with phenotypic traits. Correlations of normalized gene expression data with phenotypic traits in Iberian and Duroc pigs. AS: age at slaughter; BW: body weight; DFI: daily feed intake, BFT: backfat depth; HFT: ham fat depth; IMF_LD: intramuscular fat in the L. dorsi muscle; IMF_BF: intramuscular fat in the B. femoris muscle. Tables beside the figures include correlations significantly different from zero after multiple test correction (q < 0.05)

The correlation study was performed separately for the two breeds because of the strong effect of breed on taste receptor gene expression and the marked differences in the main phenotypic traits between both breeds [40]. The results are shown in Figs. 3 and 4. The correlation patterns differed between the two breeds. The correlation of expression values between pairs of analyzed genes (Fig. 3) revealed a predominance of positive correlations, especially in the Iberian breed and among the *TAS1R* and *TAS2R* genes, with values ranging from 0.4 to 0.6. In contrast, in the Duroc breed, these two gene families showed a variety of correlation estimates, with a negative correlation between the *TAS1R2* and *TAS1R3* genes (− 0.59), opposite to that observed in the Iberian breed (+ 0.56). Regarding fat receptors, in the Iberian population, expression of the *CD36* gene was negatively correlated with that of the *TAS1R2* and *TAS1R3* genes, which was not observed in Duroc. In contrast, in the Duroc breed, *CD36* expression was positively correlated with that of *TAS2R39*, which was not detected in the Iberian population.

When examining the correlation between gene expression and phenotypic data (Fig. 4), a few correlations remained significant after correction for multiple tests. In the Iberian breed, correlations mainly involved the *TAS1R* genes, which were correlated with intramuscular fat content in the biceps femoris muscle (*TAS1R1* and *TAS1R2*) and with the levels of certain fatty acids. None of these correlations were observed in the Duroc breed, where significant correlations were found for expression of the *GPR40* and *GPR120* genes with various fatty acids. Remarkably, in the Duroc breed, the strongest correlation was between expression of the *GPR40* gene and feed intake (− 0.77), which was absent in the Iberian breed.

### Identification of SNPs in candidate genes, genotyping, and allele frequencies of selected SNPs

SNP discovery was performed from muscle RNA-seq data obtained from 48 purebred animals. A summary of the results of the variant calling analysis is shown in Table 1.

**Table 1:** Summary of variant calling results in each breed by diet experimental group after SNP discovery using muscle RNA-seq data obtained from pure Iberian and Duroc pigs fed diets with oleic acid (HO) or carbohydrates (CH) as an energy source

Group	SNPs	INDELs	Unknown type	Total variants
Duroc CH	16,049	4059	0	20,108
Duroc HO	13,706	3712	0	17,418
Iberian CH	27,419	5749	7	33,175
Iberian HO	21,279	5074	2	29,356

A total of 122,208 SNPs and 15,635 INDELs were found at the muscle transcriptome level. The polymorphisms in the 10 evaluated candidate genes, consisting of 54 SNPs and 3 INDELs, were all found in the *TAS1R1*, *TAS1R3,* and *CD36* genes and are included in Additional file 5 Table S5. Two SNPs that showed different segregation patterns were selected for each gene. Details on the six selected SNPs, as well as the allele frequencies obtained after KASP genotyping of Iberian, Duroc, and crossbred pigs, are shown in Table 2. The six initially selected SNPs were genotyped in a subsample of 52 crossbred animals to check their informativity. The two SNPs in the *TAS1R3* gene cosegregated in the Iberian crossbred population, as did both SNPs in the *CD36* gene. For the *TAS1R1* gene, only one of the SNPs was polymorphic. Based on these observations, three SNPs, one in each gene, were selected for genotyping in the complete population of crossbred pigs and in several world-wide distributed breeds and wild boars. The observed allele frequencies are shown in Tables 2 and 3. Informativeness of the selected SNPs was limited in the Iberian crossbred population, especially for TAS1R3:c.-2126A/G (MAF = 0.026), but all three SNPs were used in the genetic association study.

**Table 2:** SNPs located in candidate genes selected for genotyping in the Iberian, Duroc, and crossbred populations

SNP	SNP location		Variant code		SNP class	Allele frequencies			
						Iberian (n = 30)	Duroc (n = 19)	Iberian x Duroc (n = 52)	Iberian x Duroc (n = 384)
TAS1R1:c.405G/C	6:67,401,628		rs338196837		Synonymous	0.000	0.447	0.019	0.075
TAS1R1:c.432C/T	6:67,401,655		rs698087274		Synonymous	0.553	1.000	1.000	
TAS1R3:c.-2269G/A	6:63,611,378		rs344677714		5’ UTR	0.000	0.472	0.010	
TAS1R3:c.-2126A/G	6:63,611,521		rs323959495		5’ UTR	0.000	0.324	0.010	0.026
CD36:c.1255 T/C	9:99,689,356		rs333516730		3’ UTR	0.617	1.000	0.847	
CD36:c.910G/T	9:99,694,876		rs80838320		Missense Arg304Ser	0.617	1.000	0.847	0.883

The allele frequencies shown correspond to the reference allele

**Table 3:** Allele frequencies in world-wide distributed breeds and wild boar populations

Breed	n	TAS1R1:c.405G/C	TAS1R3:c.-2126A/G	CD36:c.910G/T
Duroc	18	0.250	0.639	1.000
Pietrain	16	0.357	0.643	1.000
Landrace	16	0.125	0.156	1.000
Large-White	16	0.125	0.400	1.000
European wild boar	16	0.000	0.000	0.750
Asian wild boar	15	0.100	0.000	0.000

The frequencies shown correspond to the reference alleles

### Association studies

The effects of the three selected SNPs in the *TAS1R1, TAS1R3,* and *CD36* genes on phenotypic variation and on candidate gene expression were explored. Descriptive statistics of the growth, fatness, and FA composition traits are included in Additional file 2 Table S2. Numerous significant associations were found for the three analyzed SNPs and are included in Additional file 6 Table S6.

The *TAS1R1* polymorphism was significantly associated with 24 traits, including the abundance of different fatty acids, especially polyunsaturated fatty acids (PUFAs), monounsaturated fatty acids (MUFAs), and their sum, and desaturation and unsaturation indices in muscle [see Additional file 6 Table S6]. Some MUFAs in backfat and liver were also associated with the genotypes of this SNP, specifically C20:1n-9, which was observed in all tested tissues. This SNP was also associated with growth and especially fattening traits, including backfat depth at different developmental stages and intramuscular fat content. Allele TAS1R1:c.405G was associated with higher backfat, intramuscular fat, and MUFA contents in loin, liver, and backfat and with lower PUFA content in loin.

The *TAS1R3* SNP was associated with 36 traits, which were mainly related to hepatic FA composition [see Additional file 6 Table S6]. The allele TAS1R3:c.-2126A was associated with a positive effect on MUFAs and saturated fatty acids (SFAs) and a negative effect on PUFAs and n-6 content in the liver. A few associations with some MUFAs and PUFAs were also observed in backfat and loin. This allele was also associated with a negative effect on several developmental traits recorded at birth.

The SNP in the *CD36* gene was associated with 46 traits, most related to FA composition in muscle. In this tissue, the greatest effects were observed for the neutral lipid fraction, where the minor allele CD36:c.910 T was associated with positive effects on the main SFAs (C14:0, C16:0 and C18:0) and negative effects on MUFAs (C16:1n-7, C18:1n-7, C18:1n-9), PUFAs (C18:2n-6, C18:3n-3, C22:6n-3), and n-6 content. In agreement with these results, negative effects on desaturation (C18:1/C18:0; MUFA/SFA) and unsaturation indices were observed. Interestingly, a few contrasting effects were found for the polar lipid fraction of muscle in comparison to the neutral fraction (C16:1n-7, C18:2n-6, C18:3n-3). A few minority fatty acids were also affected at the level of the backfat and liver. In addition, this marker was associated with growth traits at different developmental stages (average daily gain and body weight at 150 to 215 days of age), as well as with intramuscular fat content at slaughter. Regarding these traits, the T allele was associated with a positive effect on muscle fat content and negative effects on body weight and average daily gain.

Regarding gene expression, only the SNP *CD36*:c.910G/T was associated with a significant additive effect on the expression of the *CD36* gene in the tongue papillae (p value = 1.05 × 10^–5^) of Iberian pigs, with the G allele being associated with higher expression than the T allele (GG = 0.44; GT = 0.34 and TT = 0.22). This finding is consistent with the observed breed-related effects on gene expression, since the G allele was fixed in the Duroc breed (Table 2), resulting in higher expression compared to the Iberian population, for which the T allele is present. In fact, the T allele, potentially associated with repression of *CD36* gene expression, was present only in the Iberian and wild boar populations (Tables 2 and 3).

### Effect of CD36 polymorphism on mRNA folding and protein secondary structure

**Fig. 5:**
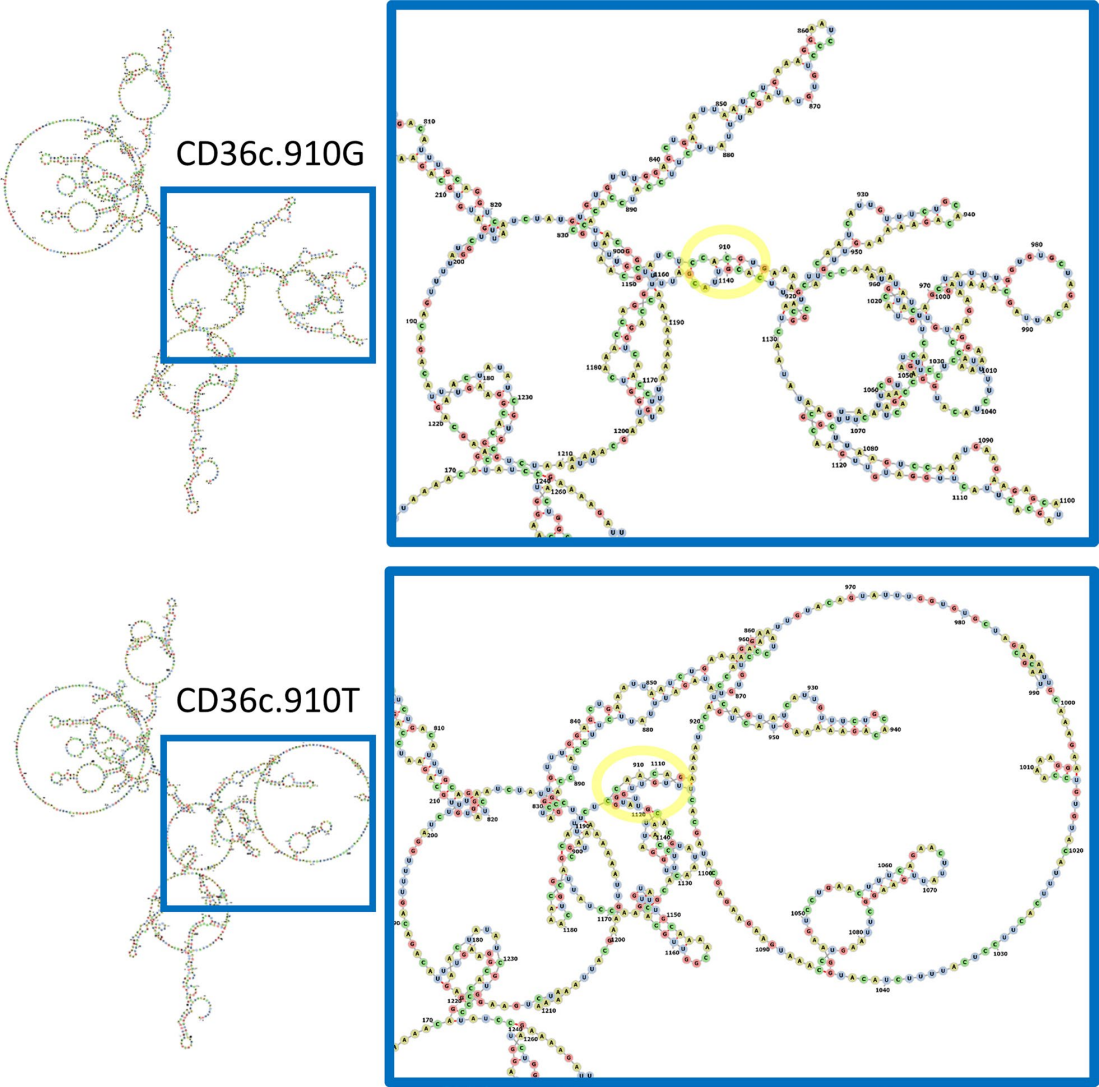
Prediction of the secondary structure of *CD36* mRNA. Secondary structure of mRNAs corresponding to both alleles of the CD36c.910G/T polymorphism, as predicted in silico with the RNAfold tool. The location of the SNP is marked with a yellow circle

We used the RNAfold tool to model the effect of the CD36c.910G/T polymorphism on mRNA secondary structure [37]. This polymorphism was predicted to produce an evident change in mRNA folding structures (Fig. 5). The estimated minimum free energy values were − 272.10 kcal/mol and − 247.40 kcal/mol for the G and T alleles, respectively. In addition, PSIPRED revealed that the aminoacidic change did not alter the secondary structure of the CD36 protein. This aminoacid mapped to a coil, which connects the more structured elements of the protein, serving as links between helices and sheets, and is essential for the protein’s overall fold and function. However, the Arg304Ser aminoacidic change did not seem to affect the coil and, consequently, the protein’s function.

### Disruption of microRNA binding sites by the CD36:c.1255 T/C SNP

The *Custom prediction* tool from miRDB [39] revealed that the CD36:c.1255C allele disrupts a binding site for the hsa-miR-181a-2-3p, which is a homologous to ssc-mir-181a-2. Presence of the C allele could prevent binding with this miRNA, inhibiting the repression of translation and/or degradation of mRNA.

## Discussion

To the best of our knowledge, only one previous study has explored the expression of taste receptor genes in the pig tongue [30]. Their work focused on characterization of genes that conform to the gustative system in pigs, and an expression study was performed in six Large White pigs to confirm the transcription of the proposed gene repertoire in the circumvallate papillae. In the present work, the effects of breed and diet on the expression of taste receptor genes in taste buds from Iberian and Duroc pigs were evaluated.

All but one of the tested genes were upregulated in the Duroc breed in comparison to the Iberian breed, although the different selected genes are involved in the perception of different tastes, with the *TAS2Rs*, *CD36,* and *GPR84* genes showing the greatest differences between breeds. The Iberian pig has traditionally been exploited in extensive systems based on seasonally available resources: stubble, crop harvest residues, and low quantities of cereals in spring and summer, and acorns fallen from oak trees and pasture in autumn and winter [41]. Downregulation of *TAS2R* genes in Iberian pigs may be linked to the dietary composition, as their traditional extensive feeding system includes vegetal resources that are rich in tannins, which have a bitter taste. In addition to the downregulated expression of the *TAS2R* genes, the expression of genes involved in the detection of sweet and umami tastes (*TAS1Rs*) and fatty acid receptors were also downregulated in Iberian pigs. These results suggest a lower general ability to detect tastes by the Iberian pig breed than by the Duroc breed, which may be consistent with a lower exigence in food selection by Iberian pigs, in agreement with their higher voluntary feed intake [2]. In fact, the higher expression of taste receptor genes in the Duroc breed is concomitant with a lower voluntary feed intake in this breed in comparison to the Iberian pigs, previously shown using these same experimental animals [40]. Results for the interaction between breed and diet also support a greater responsiveness of the Duroc breed to diet variations, as expression of three of the tested genes (*TAS1R1, TAS1R3,* and *TAS2R4*) was induced by the oleic acid content or repressed by carbohydrates, but only in the Duroc breed.

In agreement with our results, in a comparison of taste sensitivity among humans with different lifestyles, hunter gatherers showed lower overall sensitivity, as well as lower sensitivity to quinine and fructose, than their farming neighbors [42]. This lower sensitivity to different tastes could be related to the fact that a hunter-gatherer lifestyle in a forest environment relies on a diet based on a wide range of plants in order to subsist. Thus, lower taste sensitivity or exigency in food selection favors better adaptation to diverse food sources in a particular ecological niche. Iberian pigs have adapted to a traditional extensive production system known as *montanera* for centuries*,* where they graze in oak forests with variable feed resources. Repression of the taste perception pathway in Iberian pigs could be associated with higher feed intake of a large variety of foods, in agreement with their thrifty phenotype. However, additional research is needed to determine whether this represents a genetic adaptation or a consequence of other physiological and environmental factors. It should also be considered that, in addition to transcription activity, the number of taste receptors, which has not been studied in this or previous works, could also impact taste sensitivity.

In concordance with our findings, previous works have proposed that impairment of oral fat-sensing mechanisms may contribute to overeating and obesity [43–45]. Indeed, human nutritional studies have reported that obesity and a greater body mass index are associated with a reduced perceived intensity of various taste qualities and a diminished sense of taste [46] and interferes with taste bud renewal [47]. Thus, our findings indicating lower taste perception are consistent with the obese nature of the Iberian pig [8].

Interestingly, correlations of the levels of expression of taste receptor genes differed markedly between the two breeds (Figs. 3 and 4). In the Iberian pigs, the expression of genes corresponding to the *TAS1R* and *TAS2R* families were all positively correlated, suggesting a more harmonized function in this breed, despite their reduced expression in comparison to that in the Duroc breed.

Correlations between gene expression levels of taste receptor genes and key phenotypic traits also differed between the two breeds. An outstanding characteristic of the expression of taste receptor genes is its relationship with voluntary feed intake, which in turn may have consequences for many other pork production and quality traits. However, only one significant correlation was observed for this trait, with expression of the *GPR40* gene being negatively correlated with feed intake but only in the Duroc breed. This is consistent with involvement of GPR40 in satiety regulation [48], and its activation has been shown to be an effective mechanism for reducing food intake in mice [49, 50]. However, when evaluating multiple correlations, it is important to consider the possibility that some of the observed associations may be due to stochastic effects rather than true biological relationships, since there is an increased likelihood of detecting significant correlations by chance alone, although we did apply multiple test corrections for the correlation analyses. Therefore, caution should be exercised when interpreting the results and future studies should explore the potential role of this gene and its implications.

As genetic variation in taste receptors has been linked to regulation of body weight [46], a genetic association study was also carried out. First, to explore their genetic variability, a SNP discovery study from muscle RNAseq data was carried out for the same 10 taste receptor genes that were analyzed at the gene expression level.

Genotyping variants from RNA-Seq presents several technical challenges that can impact variant calling accuracy. One major limitation is allele-specific expression, which can introduce reference bias, leading to underrepresentation of alternative alleles and affecting detection of heterozygotes [51]. While GATK's joint genotyping approach (GVCF mode) improves sensitivity by integrating multiple samples, it does not directly address allele-specific expression-related biases and may miss singletons or low-frequency variants.

Although all studied genes were expressed in muscle tissue, structural variation was observed for only three genes (*TAS1R1*, *TAS1R3,* and *CD36*). Most polymorphisms were located in *CD36* gene and showed segregation only in Iberian pigs (47 out of 54 SNPs and all the three INDELs found) [Table S5], many of which were in complete linkage disequilibrium. In contrast to our findings, the work performed by da Silva et al. [30] revealed much greater variability in *TAS2R* genes than in non-bitter taste genes when analyzing 79 pig genomes representing 14 breeds or populations (including 4 Iberian and 4 Duroc animals). In fact, the *TAS2R38* and *TAS2R39* genes, which were also analyzed in the present work, were among the most polymorphic genes, while no polymorphisms were detected in these genes in our results. Nevertheless, this previous work [30] revealed that the Iberian pig was an exception to this high level of genetic variation, showing the lowest nucleotide diversity in these and other genes and a clear genetic differentiation in relation to other breeds and populations. The results of Clop et al. [16] and Muñoz et al*.* [52] also confirmed the low level of variability of these genes in Iberian and/or Duroc breeds in comparison to a wide panel of pig breeds and wild boars. The lack of variability in the *TAS2R* genes in Iberian pigs can be interpreted as a consequence of adaptation to specific feeding resources, such as acorns in the traditional *montanera* system. It has been previously proven that many and diverse selective processes have occurred in the Iberian pig lineage and among these, specifically changes in feeding behavior have been highlighted [53].

A genetic association study with phenotypic traits was performed for three SNPs in these three genes, and the results showed a relevant association of all three markers with several tissue composition measures and some growth and fattening traits. The marker located in the *TAS1R1* gene was mainly associated with fattening, as well as muscle fat content and composition, especially MUFA and PUFA content. In agreement, expression of the *TAS1R1* gene was significantly correlated with intramuscular fat content, making this gene an interesting candidate for meat quality traits. Unexpectedly, the allele TAS1R1c.405G, which is associated with positive effects on fattening and meat quality (intramuscular fat content and MUFA content in muscle), was absent in the Iberian population. As this SNP did not show relevant negative associations with growth traits and showed segregation in Iberian x Duroc crossbreds, as well as in world-wide distributed breeds, it can be considered a candidate for improving meat quality in these breeds if the identified associations are validated. The marker located in *TAS1R3* had the lowest informativeness (Minor Allelic Frequency (MAF) = 0.026) and, although main effects on fatty acid composition in the liver were observed, it should be noted that the estimated effect sizes may be biased, as only a small subset of animals carried the minor allele. In mice, allelic variation in the *TAS1R3* gene has been shown to be associated with sweet taste responsiveness and glucose metabolism [54] and the function of this gene has been related to fat accumulation and lipogenesis in the liver [55, 56]. However, associations related to fatty acid composition have not been described thus far. Interestingly, both the *TAS1R1* and *TAS1R3* markers showed significant associations with developmental traits recorded at early stages. These results are consistent with previous findings in humans that showed that *TAS1R* gene variants are associated with birth weight [57] and suggest potential interest in the development of intrauterine growth restriction processes, which are common not only in hyperprolific pigs but also in Iberian pigs [58].

The most relevant findings were observed for the missense SNP located on the *CD36* gene, which was the one with the highest informativeness (MAF = 0.12) and, moreover, showed a significant and additive association with its own transcription level of considerable magnitude (GG animals had almost twice as much expression than TT animals). The analyzed marker (rs80838320) produces an amino acid change, Arg304Ser, in the encoded protein, which was predicted to be tolerated by SIFT software (Sorting Intolerant From Tolerant, https://sift.bii.a-star.edu.sg/). In agreement with this, the PSIPRED software showed that the Arg304Ser aminoacid change did not alter the secondary structure of the CD36 protein and, therefore, its function. However, due to the clear association with gene expression, we evaluated the potential *cis*-regulatory effect of the SNP by predicting changes in the mRNA secondary structure and minimum free energy of both alleles. This in silico prediction supported an increase in free energy for allele T, which could be related to a lower stability, in agreement with its lower expression level. It should be noted, however, that the association of the SNP located in *CD36* gene with its own expression could also be due to linkage disequilibrium of this SNP with a regulatory variant.

The *CD36* polymorphism was consistently associated with different growth traits across ages, as well as with muscle fat content and composition. Allele G, which was associated with a higher expression level, was fixed in the pure Duroc breed, as well as in the Pietrain, Landrace, and Large White breeds, and was associated with higher body weight and average daily gain. The T allele, which segregates in the Iberian breed and in European wild boar and is fixed in Asian wild boar, and is associated with lower *CD36* gene expression, had positive effects on the quantity of intramuscular fat. Together, these results are in agreement with the differences in phenotype between breeds and with the lack of artificial selection in the Iberian breed. As explained before, most genetic variability detected in the studied panel of taste receptor genes was found in the *CD36* gene (47 SNPs and 3 INDELs), and many of these were in complete linkage disequilibrium with each other in the Iberian breed. Thus, the analyzed SNP is likely a marker for a haplotype block that captures most of the genetic variation in the gene.

The *CD36* gene encodes a fatty acid transporter and is a key fatty acid sensor and regulator of lipid metabolism [59]. This gene has been extensively studied in the digestive tract, especially in the intestine and liver. In addition, lingual CD36 is involved in the taste of fat, eating behavior, and obesity risk in rodents and humans [60]. In model species, variation in the *CD36* gene has been associated with body mass index, body fat, and other cardiometabolic risk factors [21, 61]. In livestock species, polymorphisms in the *CD36* gene have been recently associated with different carcass traits in chickens [62]. In pigs, this gene has been proposed to be a strong candidate gene for growth and fatness traits after observing differences in predicted allele frequencies between pigs in an F2 cross with extreme values for average daily gain and retroperitoneal fat [16]. The expression of this gene has also been evaluated in pig muscle in studies focused on understanding the effects of breed and diet on gene expression and intramuscular fat deposition [24, 63, 64]. However, to the best of our knowledge, no formal association results are available for markers in *CD36* in pig populations. Our results show a considerable effect of the analyzed SNP on relevant productive traits, with additive effects of ~ 0.5 and 0.3 to 0.4 phenotypic standard deviations for, respectively, growth traits and for intramuscular fat content and fatty acid composition. Nevertheless, its potential use in breeding programs, apparently limited to Iberian pigs, would be challenging due to its antagonistic effects on quality and growth traits.

The effects of the analyzed markers could be a consequence of the influence of taste receptors on voluntary feed intake but could also be derived from peripheral effects in other nonoral tissues, where all evaluated genes are known to have complementary roles in metabolism regulation and body homeostasis [65, 66]. Unfortunately, we do not have records of individual feed intake for the crossbred population that was employed for the association analysis and, thus, biological mechanisms related to variability in appetite cannot be inferred.

For the three analyzed markers, allele frequencies observed in pure Iberian animals were similar to those observed in European wild boar, in agreement with the genetic closeness observed between this rustic and scarcely selected breed and its ancestor [67]. In contrast, these allele frequencies differed from those observed for world-wide distributed breeds, for which the *TAS1R1* and *TAS1R3* markers showed segregation, while the *CD36* polymorphism segregated only in Iberian populations. Notably, the allele frequencies of SNPs in the *TAS1R1* and *TAS1R3* genes were quite different between the two analyzed Duroc populations. This finding may be related to within-breed genetic heterogeneity [68] and the different origins of these two Duroc populations, as the Duroc pigs employed for the gene expression study had a French origin, but those employed for allele frequency estimation covered several different populations across the Iberian Peninsula, representative of Spanish Duroc pigs.

## Conclusions

Most of the taste receptor genes analyzed in this study, which correspond to different taste perceptions, showed lower expression levels and responses to diet in taste buds of Iberian compared to those of Duroc growing pigs. This aligns with lower overall taste sensitivity of Iberian pigs, their limited feed selection, and the obese phenotype and thrifty metabolism characteristic for this local rustic breed.

Structural variation in the selected genes was limited in both breeds and was concentrated in a few genes, particularly in the *CD36* gene. Genetic markers in the *TAS1R1* and *TAS1R3* genes showed segregation in several world-wide distributed breeds and were mainly associated with meat quality and liver composition in Duroc x Iberian crossbred pigs. The missense variant in the *CD36* gene*,* which segregated in the Iberian pig population, was associated with relevant growth and tissue composition traits, as well as with the expression level of the *CD36* gene in the circumvallate papillae of Iberian pigs. A potential *cis*-acting regulatory role is proposed for this variant, considering the predicted lower mRNA stability of the mutant allele.

## Supplementary Information

Additional file 1: Table S1. Experimental diets. Ingredient composition and estimated and analyzed composition of the diets (per kg diet).

Additional file 2: Table S2. Descriptive statistics of the phenotypic traits employed for the genetic association study. Mean, standard deviation, minimum and maximum values for each analyzed phenotypic trait, grouped in two Excel sheets: growth and fatness (weight, morphological measures and backfat at different time points during development as well as carcass traits at slaughter), fatty acid composition (in backfat, l. dorsi muscle and liver).

Additional file 3: Table S3. RT‒qPCR primer details for the porcine nutrient sensing and taste receptor gene repertoire. Sequence of qPCR primers and size of the amplified fragments for the 10 selected taste receptor genes (*TAS1R1, TAS1R2, TAS1R3, TAS2R4, TAS2R38, TAS2R39, GPR120, GPR40, GPR84* and *CD36*) and 2 endogenous genes employed for normalization of gene expression data (*GAPDH* and *ACTB*).

Additional file 4: Table S4. Fixed effects and covariates. Effects included in the model for the association analyses of the different groups of traits.

Additional file 5: Table S5. List of SNPs and INDELs detected in taste receptor genes. Chromosomal position, alleles and allele frequencies in the Iberian and Duroc populations of the 54 SNPs and 3 INDELs detected from muscle RNAseq data in the selected panel of taste receptor genes. The SNPs selected for genotyping are highlighted in yellow.

Additional file 6: Table S6. Results of a genetic association study of growth, fatness, carcass and fatty acid composition traits with SNPs located in the *TAS1R1*, *TAS1R3* and *CD36* genes. Significant association results are included (p < 0.05) for the three analyzed SNPs: *TAS1R1*:c.405G/C, *TAS1R3*:c.-2126A/G and *CD36*:c.910G/T. Positive effects correspond to the minor allele. One Excel sheet is included for each SNP.

## Data Availability

All data generated or analyzed during this study are included in this published article and its supplementary information files.
