# Supplementary material for: Expression and structural analysis of taste receptor genes in Iberian and Duroc pigs
Source: Genet Sel Evol. 2025 May 2;57:22. doi: 10.1186/s12711-025-00968-0 (PMC12046925; doi:10.1186/s12711-025-00968-0)
Supplement: Supplementary file 3 — Additional file 3: Table S3. RT‒qPCR primer details for the porcine nutrient sensing and taste receptor gene repertoire. Sequence of qPCR primers and size of the amplified fragments for the 10 selected taste receptor genes (TAS1R1, TAS1R2, TAS1R3, TAS2R4, TAS2R38, TAS2R39, GPR120, GPR40, GPR84 and CD36) and 2 endogenous genes employed for normalization of gene expression data (GAPDH and ACTB). [file 12711_2025_968_MOESM3_ESM.docx]

| Gene | Forward primer 5’-3’ | Reverse primer 5’-3’ | Size (bp) |
| --- | --- | --- | --- |
| *Tas1r1* | ATCTGTTCTCGAGGCCAAGTCT | GCGAGTCCCCACTGTCACTAA | 109 |
| *Tas1r2* | GCCACCATGACCGTGGCCCACTT | AGGTCCTCACTGATGGCACTGTAGC | 77 |
| *Tas1r3* | CGCAGCATTGCCACCTACTG | TAGCTGACCTGCGGCATGAG | 134 |
| *Tas2r4* | GAGGGCATCTTGTCCTTGCT | GAGCTTCAGTCTGGGGGTTC | 160 |
| *Tas2r38* | CAGCCTGGAGGCCCATTTTA | AGGCTGCCAGTATCCCTACA | 138 |
| *Tas2r39* | CCTTGGGCCATAGTGGACTC | CATGTGCTGGGTGTGTCTCT | 182 |
| *GPR120* | GATTTGGCCCAGTGTTGCTG | TCTGGTGGCTCTCGGAGTAA | 170 |
| *GPR40* | GCCTAGGACCCTACAATGCC | GATGGGCCCCCTTTTGTTCT | 187 |
| *GPR84* | CTCATCGCCAACCTCACAGT | GGAGACAGAGTTGGACGCAA | 150 |
| *CD36* | ATGCAAAGAAGGAAAACCCGTGTA | GTAGCCGTTGTGCAAATCGTAAAG | 167 |
| *GAPDH* | TGGTGAAGGTCGGAGTGAAC | GAAGGGGTCATTGATGGCGA | 104 |
| *ACTB* | TCTGGCACCACACCTTCT | GATCTGGGTCATCTTCTCAC | 153 |
